# Supplementary material for: ZeroBind: a protein-specific zero-shot predictor with subgraph matching for drug-target interactions
Source: Nat Commun. 2023 Nov 29;14:7861. doi: 10.1038/s41467-023-43597-1 (PMC10687269; doi:10.1038/s41467-023-43597-1)
Supplement: Supplementary file 1 — SupplementaryInformation [file 41467_2023_43597_MOESM1_ESM.pdf]

ZeroBind: A protein-specific zero-shot predictor with subgraph matching for drug-target interactions

Yuxuan Wang[1], Ying Xia[1], Junchi Yan[2], Ye Yuan[1], Hong-Bin Shen[1], Xiaoyong Pan[1]*

[1]Institute of Image Processing and Pattern Recognition, Shanghai Jiao Tong University, and Key Laboratory of System Control and Information Processing, Ministry of Education of China, Shanghai 200240, China.

[2]Department of Computer Science and Engineering, and MoE Key Lab of Artificial Intelligence, AI Institute, Shanghai Jiao Tong University, Shanghai, 200240, China

*Corresponding authors: X. Pan, 2008xypan@sjtu.edu.cn

Supplementary information

Supplementary Table 1. Zero-shot performance evaluation with ZeroBind and baseline methods on three independent test sets. The average is reported after performing each experiment five times, along with the standard deviation. The bold face indicates the method is the best across the compared methods.

Metric	Method	Transductive test	Semi-inductive test	Inductive test
AUROC	DeepConv-DTI	0.8831±0.0051*	0.7541±0.0049*	0.6973±0.0157*
	GraphDTA	0.9122±0.0042*	0.7559±0.0041*	0.7282±0.0124*
	Deeppurpose	0.9035±0.0081*	0.7875±0.0072*	0.7873±0.0071*
	AI-bind	0.9156±0.0047*	0.7453±0.0126*	0.7868±0.0048*
	DrugBAN	0.9256±0.0046*	0.7871±0.0047*	0.7584±0.0072*
	**ZeroBind**	**0.9521±0.0034**	**0.8681±0.0052**	**0.8139±0.0035**
AUPRC	DeepConv-DTI	0.9686±0.0062*	0.9524±0.0102*	0.9386±0.0042*
	GraphDTA	0.9654±0.0035*	0.9691±0.0034*	0.9573±0.0032*
	Deeppurpose	0.9798±0.0027*	0.9764±0.0048*	0.9721±0.0049*
	AI-bind	0.9653±0.0051*	0.9786±0.0053*	0.9754±0.0031*
	DrugBAN	0.9763±0.0048*	0.9640±0.0016*	0.9615±0.0088*
	**ZeroBind**	**0.9896±0.0018**	**0.9880±0.0041**	**0.9872±0.0037**

*P-value of two-sided t-test is <0.05

Supplementary Table 2. Few-shot performance evaluation of ZeroBind with baseline methods on the few-shot test sets. The average is reported after performing each experiment five times, along with the standard deviation. Two-sided The bold face indicates the method is the best across the compared methods

Method	Few-shot test set	
	AUROC	AUPRC
DeepConv-DTI	0.8973±0.0162*	0.9431±0.0149*

GraphDTA	0.9391±0.0131*	0.9675±0.0148*
Deeppurpose	0.9545±0.0063*	0.9487±0.0095*
AI-bind	0.9572±0.0125*	0.9418±0.0148*
DrugBAN	0.9114±0.0088*	0.9281±0.0052*
**ZeroBind**	**0.9721±0.0068**	**0.9832±0.0048**

*P-value of two-sided t-test is <0.05

Supplementary Table 3. The protein names and the PDB IDs used in COVID datasets.

Protein name	PDB ID
nucleocapsid protein N-terminal RNA binding domain	6M3M
ORF7A encoded accessory protein	6W37
u1S2q 1-RBD Up Spike Protein Trimer	6X2A
ORF3a	6XDC
main protease	6Y2E
Orf9b	6Z4U
ORF8 accessory protein	7JTL
Main Protease (Mpro) H172Y Mutant	8D4J
