# Supplementary material for: ZeroBind: a protein-specific zero-shot predictor with subgraph matching for drug-target interactions
Source: Nat Commun. 2023 Nov 29;14:7861. doi: 10.1038/s41467-023-43597-1 (PMC10687269; doi:10.1038/s41467-023-43597-1)
Supplement: Supplementary file 3 — Reporting Summary [file 41467_2023_43597_MOESM3_ESM.pdf]

nature portfolio

Corresponding author(s): Xiaoyong Pan

Last updated by author(s): Oct 15, 2023

Reporting Summary

Nature Portfolio wishes to improve the reproducibility of the work that we publish. This form provides structure for consistency and transparency in reporting. For further information on Nature Portfolio policies, see our Editorial Policies and the Editorial Policy Checklist.

Statistics

For all statistical analyses, confirm that the following items are present in the figure legend, table legend, main text, or Methods section.

n/a	Confirmed	
☐	☒	The exact sample size (*n*) for each experimental group/condition, given as a discrete number and unit of measurement
☒	☐	A statement on whether measurements were taken from distinct samples or whether the same sample was measured repeatedly
☐	☒	The statistical test(s) used AND whether they are one- or two-sided<br>*Only common tests should be described solely by name; describe more complex techniques in the Methods section.*
☒	☐	A description of all covariates tested
☒	☐	A description of any assumptions or corrections, such as tests of normality and adjustment for multiple comparisons
☐	☒	A full description of the statistical parameters including central tendency (e.g. means) or other basic estimates (e.g. regression coefficient) AND variation (e.g. standard deviation) or associated estimates of uncertainty (e.g. confidence intervals)
☐	☒	For null hypothesis testing, the test statistic (e.g. *F*, *t*, *r*) with confidence intervals, effect sizes, degrees of freedom and *P* value noted<br>*Give P values as exact values whenever suitable.*
☒	☐	For Bayesian analysis, information on the choice of priors and Markov chain Monte Carlo settings
☐	☒	For hierarchical and complex designs, identification of the appropriate level for tests and full reporting of outcomes
☒	☐	Estimates of effect sizes (e.g. Cohen's *d*, Pearson's *r*), indicating how they were calculated

Our web collection on statistics for biologists contains articles on many of the points above.

Software and code

Policy information about availability of computer code

Data collection	No commercial software is used in this study. BindingDB is a public database of DTI interactions, which deposits binding affinity data between drugs (drug-like molecules) and target proteins. It currently contains over 2,600,000 experimentally determined binding affinities of protein-drug complexes between over 8,000 protein targets and over 1,100,000 small molecules. The protein structures are collected from PDB database and the predicted protein structures are collected from the https://alphafold.com/. we download the true binding pocket information containing pocket residuals and pocket 3D coordinate locations from the PDBbind database, which contains binding pockets for 14,336 DTIs.
Data analysis	ZeroBind is a computational method for drug-target interaction prediction and all software are given at https://github.com/mypreciousshh/ZeroBind. The experiments are performed using Pytorch v2.0.0, torchvision v0.15.0, torchaudio v2.0.0, pyg v2.3.0, lightning v2.0.1, graphein v 1.4.0, fair-esm v 2.0.0

For manuscripts utilizing custom algorithms or software that are central to the research but not yet described in published literature, software must be made available to editors and reviewers. We strongly encourage code deposition in a community repository (e.g. GitHub). See the Nature Portfolio guidelines for submitting code & software for further information.

Data

Policy information about availability of data

All manuscripts must include a data availability statement. This statement should provide the following information, where applicable:

- Accession codes, unique identifiers, or web links for publicly available datasets
- A description of any restrictions on data availability
- For clinical datasets or third party data, please ensure that the statement adheres to our policy

Data availability
The online webserver is freely available at http://www.csbio.sjtu.edu.cn/bioinf/ZeroBind/.
The benchmark dataset is collected from the original database BindingDB and available at http://www.csbio.sjtu.edu.cn/bioinf/ZeroBind/datasets.html along with the SARS-COV-2 test dataset, and the experimental protein structure data used in this study are downloaded from the RCSB PDB database https://www.rcsb.org/downloads/ and the predicted structures by AlphaFold are downloaded from AlphaFold Protein Structure Database https://www.alphafold.ebi.ac.uk/.

Research involving human participants, their data, or biological material

Policy information about studies with human participants or human data. See also policy information about sex, gender (identity/presentation), and sexual orientation and race, ethnicity and racism.

Reporting on sex and gender	This is no relevant to our study.
Reporting on race, ethnicity, or other socially relevant groupings	This is no relevant to our study.
Population characteristics	This is no relevant to our study.
Recruitment	This is no relevant to our study.
Ethics oversight	This is no relevant to our study.

Note that full information on the approval of the study protocol must also be provided in the manuscript.

Field-specific reporting

Please select the one below that is the best fit for your research. If you are not sure, read the appropriate sections before making your selection.

☒ Life sciences ☐ Behavioural & social sciences ☐ Ecological, evolutionary & environmental sciences

For a reference copy of the document with all sections, see nature.com/documents/nr-reporting-summary-flat.pdf

Life sciences study design

All studies must disclose on these points even when the disclosure is negative.

Sample size	The database currently contains over 2,600,000 experimentally determined binding affinities of protein-drug complexes between over 8,000 protein targets and over 1,100,000 small molecules. These benchmark datasets are widely used in published methods and the data is sufficient for evaluating the proposed method and baseline methods since the datasets provide sufficient number of data samples for training-test set split and repeating the experiments multiple times to calculate the significance value.
Data exclusions	First, data points are filtered with “single protein” for the “target type” attribute, and kinetic constants K_i, K_d, IC_50 and EC_50 for the “standard type” attribute. In addition, all target proteins should be human or human-like proteins, so they are filtered using "Homo sapiens" for "Target Source Organism" attribute. After excluding proteins that don’t have SwissProt name and molecules that cannot be handled by RDKit 37, 1,500,000 protein-drug pairs were collected. This is done to ensure that the proteins and molecules are functional data.
Replication	The experiments in this study were repeated five times with different random seeds
Randomization	We first use cd-hit, a widely used program for clustering protein sequences, to cluster 1603 proteins into 1101 clusters with a similarity threshold 0.4. Through comparing molecules scaffolds, we split the molecules into the training molecule set and test molecule set with a training ratio of 0.95, and ensure that these two sets do not have overlapping scaffolds. As the meta-learning-based framework requires sufficient data, we first divide the clusters which have any protein with the number of associated molecules less than 20 into the test clusters, and the remaining clusters as the training clusters. Then, we construct the training set using 95% of the proteins in the training clusters and SMILES in the training molecules set. The remaining 5% of the proteins in the training clusters and smiles in the training molecules set are used as the Transductive test set. Finally, the proteins and smiles both in the test clusters and molecule set are further used as the Inductive test set, and the rest data are used as the Semi-inductive test set.
Blinding	For zero-shot prediction, our method does not see the proteins and molecules as blinding test during the model training, where blinding test is evaluated in the inductive test set. In addition, the prediction results for SARA-COV-2 validation the blinding test.

Reporting for specific materials, systems and methods

We require information from authors about some types of materials, experimental systems and methods used in many studies. Here, indicate whether each material, system or method listed is relevant to your study. If you are not sure if a list item applies to your research, read the appropriate section before selecting a response.

Materials & experimental systems

n/a	Involved in the study
☒	☐ Antibodies
☒	☐ Eukaryotic cell lines
☒	☐ Palaeontology and archaeology
☒	☐ Animals and other organisms
☒	☐ Clinical data
☒	☐ Dual use research of concern
☒	☐ Plants

Methods

n/a	Involved in the study
☒	☐ ChIP-seq
☒	☐ Flow cytometry
☒	☐ MRI-based neuroimaging
